# Ovine Carotid Artery-Derived Cells as an Optimized Supportive Cell Layer in 2-D Capillary Network Assays

**DOI:** 10.1371/journal.pone.0091664

**Published:** 2014-03-12

**Authors:** Stefan Weinandy, Patrick Babczyk, Agnieszka Dreier, Ronald E. Unger, Thomas C. Flanagan, C. James Kirkpatrick, Martin Zenke, Doris Klee, Stefan Jockenhoevel

**Affiliations:** 1 Department of Tissue Engineering & Textile Implants, AME - Helmholtz Institute for Biomedical Engineering, RWTH Aachen University, Aachen, Germany; 2 Department of Neurosurgery, Medical Faculty, RWTH Aachen University, Aachen, Germany; 3 Institute of Pathology, University Medical Center, Johannes Gutenberg University of Mainz, Mainz, Germany; 4 School of Medicine & Medical Science, Health Sciences Centre, University College Dublin, Dublin, Ireland; 5 Department of Cell Biology, Institute for Biomedical Engineering, RWTH Aachen University, Aachen, Germany; 6 Institute of Technical and Macromolecular Chemistry (ITMC), RWTH Aachen University, Aachen, Germany; Georgia Regents University, United States of America

## Abstract

**Background:**

Endothelial cell co-culture assays are differentiation assays which simulate the formation of capillary-like tubules with the aid of a supportive cell layer. Different cell types have been employed as a supportive cell layer, including human pulmonary artery smooth muscle cells (PASMCs) and human mammary fibroblasts. However, these sources of human tissue-derived cells are limited, and more readily accessible human or animal tissue-derived cell sources would simplify the endothelial cell co-culture assay. In the present study, we investigated the potential use of alternative, accessible supportive cells for endothelial cell co-culture assay, including human umbilical cord and ovine carotid artery. *Methods* *and Results*: Human umbilical artery SMCs (HUASMCs) and ovine carotid artery-derived cells were seeded into 96-well plates, followed by addition of human umbilical vein endothelial cells (HUVECs). Nine days after co-culture, cells were fixed, immunostained and analysed using an *in vitro* angiogenesis quantification tool. Capillary-like structures were detected on ovine carotid artery-derived supportive cell layers. The initial cell number, as well as pro- and anti-angiogenic factors (VEGF, PDGF-BB and Bevacizumab), had a positive or negative influence on the number of capillary-like structures. Furthermore, HUVECs from different donors showed distinct levels of VEGF receptor-2, which correlated with the amount of capillary-like structures. In the case of HUASMC supportive cell layers, HUVECs detached almost completely from the surface.

**Conclusions:**

Cells of different origin have a varying applicability regarding the endothelial cell co-culture assay: under the conditions described here, ovine carotid artery-derived cells seem to be more suitable than HUASMCs for an endothelial co-culture assay. Furthermore, the ovine carotid artery-derived cells are easier to obtain and are in more abundant supply than the currently used dermal or breast tissue cells. The use of ovine carotid artery-derived cells simplifies the endothelial co-culture assay with respect to testing large amounts of pro- and anti-angiogenic factors.

## Introduction

The blood vessel system is essential for supplying cells with nutrients and oxygen, in addition to the removal of waste products. All cells in an organ are located in close proximity to this supportive system (within ∼100–200 μm, which is the limit of oxygen diffusion). To influence vessel growth and ensure their own sustenance, tumour cells release growth factors such as vascular endothelial growth factor (VEGF), leading to tumour-directed vessel development [1]. This process is called pathological angiogenesis, a development of new vessels from pre-existing vessels. Different angiostatic drugs (e.g. angiostatin or bevacizumab) [2], [3] can be applied to disrupt vessel growth and therefore limit tumour nutrition. Different *in vitro* and *in vivo* assays have been developed to investigate the effect of angiostatic drugs.

A commonly used and well-known assay is the Matrigel assay, which is rather poorly characterized. Capillary-like structures with lumen have been described using this assay, although there is significant debate as to whether these structures actually contain patent lumina [4] or not [5]. Furthermore, non-endothelial cells such as fibroblasts and other cell types also form tubules on Matrigel [6]. For this reason, the results need to be interpreted with caution and more than one assay should be taken into consideration.

A further assay used to evaluate the efficacy of angiostatic drugs is the endothelial cell co-culture assay. This assay is based on a supportive mural cell layer, on which endothelial cells have the ability to form capillary-like structures after 7–14 days [7]. Although this assay takes longer than the Matrigel assay (4–6 h), it provides a more physiological environment, with tubules growing on the supportive mural cell layer matrix. For the endothelial cell co-culture assay, a variety of different cell types have been employed as a supportive cell layer, including pulmonary artery smooth muscle cells (PASMCs) [8], primary human mammary fibroblasts [6] and human dermal fibroblasts (DF) [9]. However, these sources of human tissue-derived cell are limited, and more accessible human or animal tissue-derived cell sources would be an advantage for endothelial cell co-culture assays.

In the present approach, HUASMCs and ovine carotid-artery derived cells were investigated as accessible, supportive cell layers for endothelial cell co-culture assays. We evaluated the influence of cell numbers within the supportive cell layer, in addition to that of pro-angiogenic factors (PDGF-BB, VEGF-A) and anti-angiogenic factors (Bevacizumab), on vessel development. The presence of VEGF receptor-2 (VEGFR-2) on the employed HUVEC cell lines as the tubule-forming units was also evaluated to determine any correlation with the amount of capillary-like structures formed *in vitro*.

## Methods

### Cell culture

The sheep cadavers used to isolate the cells for these experiments were kindly provided by the Institute for Laboratory Animal Science and Experimental Surgery, RWTH Aachen University. Cadavers were obtained from animal studies which were conducted and approved previously by the local ethical committee NRW (87-51.04.2010.A351). All animals were treated in compliance with the European Convention on Animal Care.

**Figure 1:**
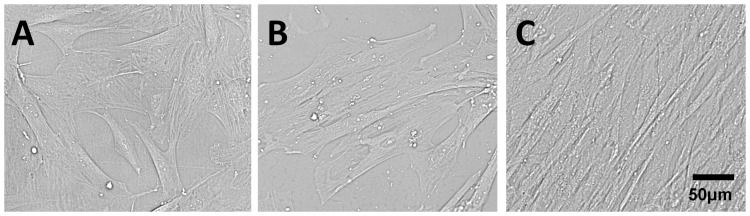
Morphology of supportive cells. (A) carotid artery-derived mixed population of cells (P4); (B) subconfluent HUASMCs and (C) confluent HUASMCs with their typical hill and valley morphology

**Figure 2:**
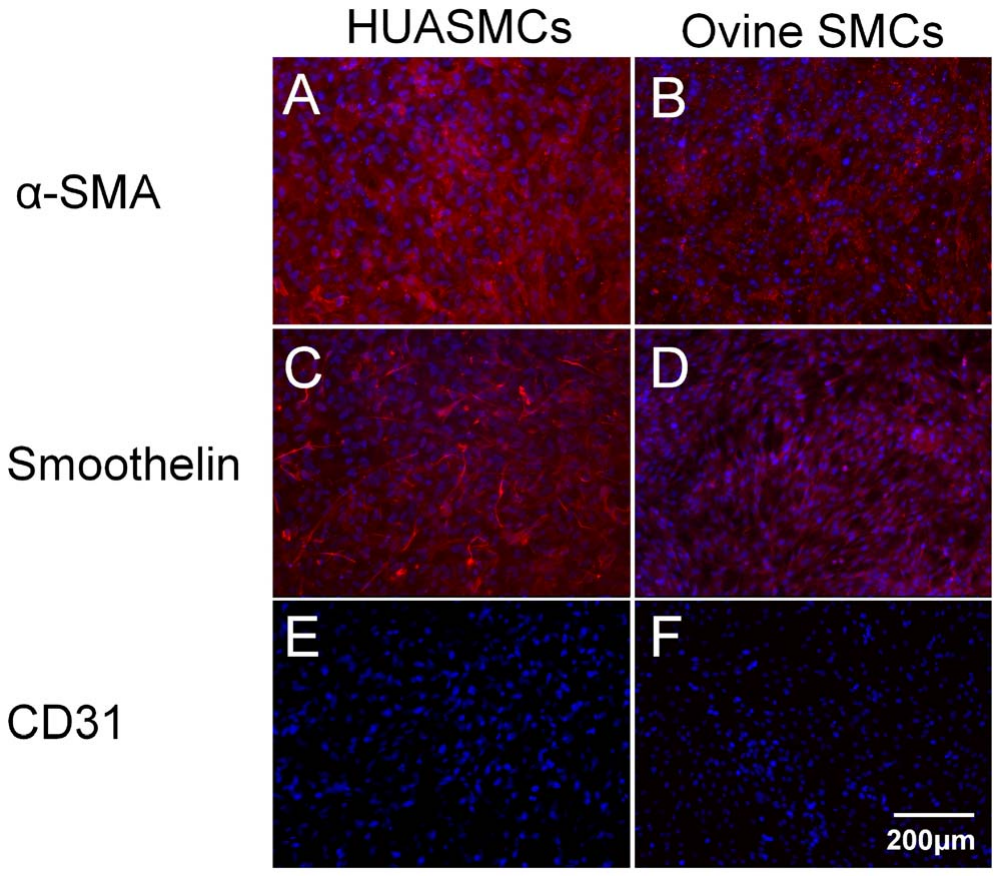
Characterization of supportive cells. Immunhistological staining of (A–C) human umbilical artery smooth muscle cells and (D–F) ovine carotid artery smooth muscle cells for (A, B) α-SMA (red), (C, D) smoothelin (red) and (E, F) CD31, (DAPI staining in blue)

For isolation of ovine cells, carotid arteries from juvenile sheep cadaver were harvested under sterile surgical conditions. The carotid arteries were washed with phosphate-buffered saline (PBS; PAA Laboratories GmbH, Pasching, Austria) before being minced into 1-mm rings. The tissue pieces were bathed in standard medium (Dulbecco's Modified Eagle's Medium (DMEM; Gibco, Karlsruhe, Germany) +10% foetal bovine serum (FBS; PAA) in tissue culture flasks for primary explant culture, and transferred to a humidified incubator at 37 °C and 5% CO_2_. Serially passaging of the resulting population of cells was achieved using 0.25% trypsin/0.02% ethylenediaminetetraacetic acid solution (PAA)). The morphology of the cells after 2 days of culture in 96-well plates is depicted in Figure 1A. The cells were stained positively for alpha smooth muscle actin (α-SMA) (Sigma, Seelze, Germany) and smoothelin (Abcam, Cambridge, UK) (Figure 2). In addition, the endothelial cell marker, CD31 (Sigma+Dako, Hamburg, Germany), demonstrated an absence of endothelial cells from the cultures (Figure 2).

Umbilical cord-derived cells were obtained in accordance with the human subjects approval of the medical faculties' local ethics committee at RWTH Aachen University (votum of the local ethics committee: #EK 2067). The approval includes the use of umbilical cord-derived cells for blood vessel development *in vitro*. Participants provided their written consent to participate in this study. Umbilical cords were kindly provided by the Department of Gynaecology at the University Hospital in Aachen.

For isolation of human umbilical artery smooth muscle cells (HUASMCs), umbilical arteries were processed as described above for carotid arteries. HUASMCs were cultured in a petri dish (Figure 1B) and exhibited a typical “hill and valley” morphology after 2 days of culture in 96-well plates (Figure 1C). The isolated cells were characterised as smooth muscle cells based on positive staining for α-SMA and smoothelin as above (Figure 2). The isolated cell population was cultivated and passaged in an identical manner to the carotid artery-derived cells.

Endothelial cells (HUVECs) were isolated from human umbilical vein using 1 mg/ml collagenase (Type II; Gibco). Cells were re-seeded on 2% gelatin-pre-coated (Sigma-Aldrich) tissue culture flasks (T-75; Greiner, Kremsmünster, Germany) in EGM-2 medium (Lonza, Cologne, Germany) as supplemented according to the manufacturer. The isolated cell population was positively stained for CD31 as an endothelial cell marker. Furthermore, the cells were observed to grow with the typical cobblestone morphology of endothelial cells.

### Endothelial co-culture assay

**Figure 3.Quantification:**
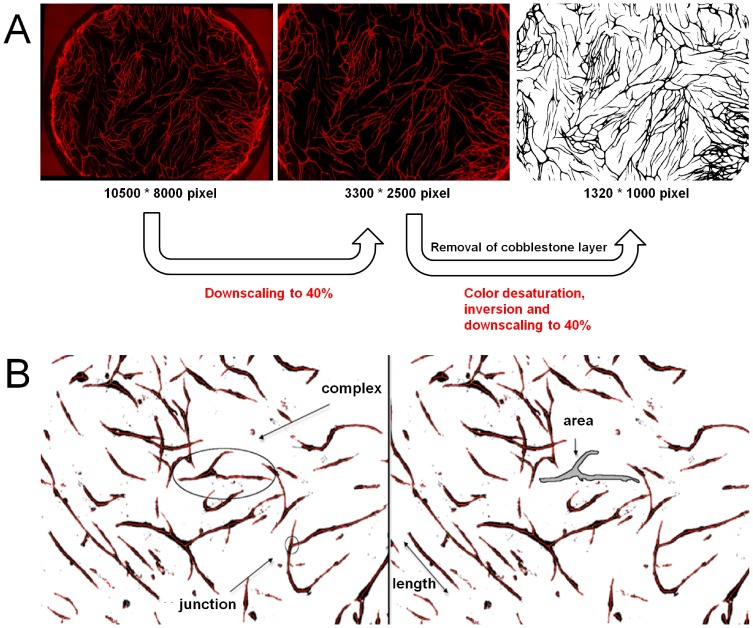
of capillary-like structures in the co-culture system. (A) Pre-processing of the obtained mosaix images for analysis in Angioquant, (B) explanation of length, junctions, area and complex as defined in Angioquant, CD31 staining (endothelial cells, red)

Supportive cells (passage 4) and HUVECs (passage 3) were employed together in the co-culture assay. The assay was performed by seeding 1−1.75×10^4^ supportive cells in DMEM +10% FBS into gelatin-pre-coated 96-well plates. After 48 h, medium was exchanged with a HUVEC suspension in EGM-2 medium (1×10^4^ cells/ml). The medium was changed every 3–4 days with addition of different growth factors, namely platelet-derived growth factor (PDGF-BB; 5–15 ng/ml; Immunotools, Friesoythe, Germany) and vascular endothelial growth factor (VEGF-A165; 30–40 ng/ml; Biomol, Hamburg, Germany). For anti-angiogenic investigation, Bevacizumab (Avastin; Roche, Basel, Switzerland) was added to the wells (2 mg/ml). On day 11, cells were fixed with methanol (−20°C) and immunostained for PECAM-1 (Sigma Aldrich) and DAPI nuclear stain (Carl Roth, Karlsruhe, Germany). The resultant capillary-like structures were quantified by imaging the entire 96-wells (mosaix image; AxioVision Software 4.8; Zeiss, Jena, Germany), defining the maximum region of interest in the well centre (width: 3300 pixels; height: 2470 pixels), and analysing with Angioquant Software [10]. This freely-available image analysis software measures length, number of complexes, number of branch points and covered area of vessels. Before analysis, images were required to be converted to negative and greyscale format. Furthermore, the images were resized to 40% twice due to file size limits (Figure 3). For image analysis, batch function in Angioquant was used. At least n = 3 wells per condition were analysed. The programme settings were: Separate Thresholds: No (same); Automatic Threshold: Yes; Kernel: 1; Remove edge tubes: No; Prune size: 3. The resulting csv-files were converted to a bar graph. The experiment was performed in triplicate with endothelial cells and supportive cells from different donors (data not shown).

### Immunohistochemistry

CD31, α-SMA (Sigma) and smoothelin (Abcam) primary antibodies (Sigma) were diluted (1∶400) in blocking buffer. 100 μl of the diluted antibody was added to each of the wells (1 h; 37°C). After washing with blocking buffer, 100 μl of AlexaFluor594-conjugated goat-anti-mouse secondary antibody was added for 1 h at 37°C (1∶400; Molecular Probes, Darmstadt, Germany). The cell nuclei were counterstained using DAPI nucleic acid stain (Molecular Probes). As negative controls, samples were incubated only in diluent and secondary antibody. Endothelial cells served as a positive control. All stained wells were viewed using an epifluorescence microscope (AxioObserver; Carl Zeiss). Images were acquired using a monochromatic digital camera (MRm; Carl Zeiss) and processed using digital software (AxioVision 4.8; Carl Zeiss).

### Flow cytometric analysis

500,000 HUVECs were resuspended in 1 ml blocking buffer (1% BSA in PBS) and incubated at 4°C to block unspecific binding sites (30 min). After centrifugation (500 g, 5 min), fluorescently-labeled VEGFR-2 antibody (Allophycocyanine dye; R&D Systems, Wiesbaden, Germany) or isotype control (R&D Systems) was added and incubated for 1 h at 4°C. Cells were then washed three times by centrifugation and resuspended in buffer (0.1% BSA in PBS) for flow cytometric analysis (FACS Canto II, BD, Heidelberg).

### Immunoprecipitation

Co-cultures were seeded in 6well plates in comparable cell amounts with respect to the 96well plates with a high (1×10∧6 per cell type) and a low seeding density (5×10∧5 per cell type). Cells were seeded as described for the endothelial co-culture assay. One day after HUVEC cell seeding, medium was exchanged with EGM-2 endothelial medium without VEGF 2 h before VEGF stimulation. VEGF (40 ng/mL) has been added to the culture for 5 min according to the literature (13). To demonstrate the correlation between VE-Cadherin and phosphorylated VEGFR-2, VE-Cadherin antibody has been added to the co-culture 1 h prior to VEGF stimulation in another condition. The control has been treated in the same way without the addition of VEGF or VE-Cadherin.

After stimulation with VEGF, cells were extracted in RIPA lysis buffer, as described in detail by Dreier et al.[11]. Protein content was measured and supernatants containing 500 μg protein were incubated over night at 4°C with 10 ug anti-VEGFR-2 antibody (Novus Biologicals, Littleton, USA). The immune complexes were captured by adding 25 μl of protein G-Sepharose (50% v/v) (GE Healthcare) and incubated for 3 h at 4°C. Immune complexes were washed three times with lysis buffer, resuspended in Laemmli sample buffer, boiled for 5 min and. run in SDS-PAGE (7,5% polyacrylamide) for blot with anti-phosphotyrosine (PY20; Santa Cruz). The remaining lysates were run in parallel and blotted with anti-α-Tubulin antibody to show equal loading.

## Statistics

The endothelial co-culture assay was developed using different supportive ovine cell lines (n = 6). All experiments were performed in triplicate, with quantification values corresponding to the mean values of three different wells. Student's t-test was performed for standard deviation errors.

## Results

### Ovine carotid artery- and human umbilical artery-derived cell characterization

Both isolated cell populations from ovine carotid artery and human umbilical artery were characterised as smooth muscle cell populations based on positive staining for α-SMA and smoothelin, and an absence of CD31-positive cells in the cultures.

### Endothelial co-culture assay

HUVECs were observed to form capillary-like structures on all carotid artery-derived cells originating from different donor sheep (n = 6). In contrast, no capillary-like structures were observed in HUVECs cultured on a HUASMC supportive layer. The amount of capillary-like structures on carotid-artery derived cells was dependent on the addition of growth factors and the number of seeded cells.

**Figure 4:**
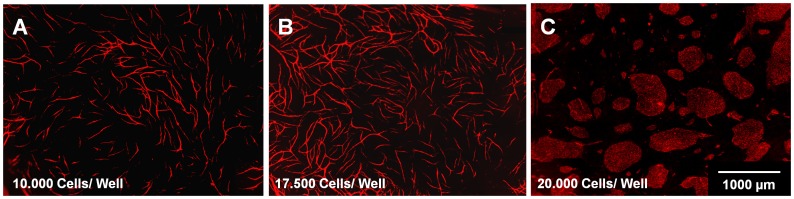
Evaluation of cell number of both supportive and vessel-forming cells in the co-culture assay. (A) 10,000 cells/well, (B) 17,500 cells/well and (C) 20,000 cells/well for each cell type; CD31 staining (endothelial cells, red)

Regarding the cell number, 1.75×10^4^ supportive and endothelial cells resulted in the most extensive vessel networks with regard to length, number of junctions and area. Co-cultures with fewer cell numbers (1×10^4^ per cell type) formed less extensive structures, while co-cultures with larger cell numbers (2×10^4^ per cell type) promoted the formation of mats of endothelial cells, rather than vessel structures (Figure 4).

**Figure 5:**
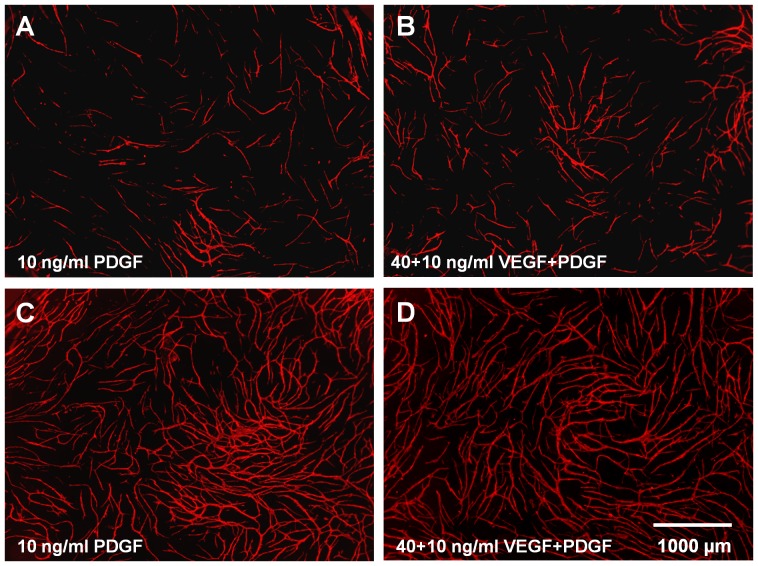
Effect of PDGF-BB and VEGF on HUVEC donor 1. Addition of PDGF-BB and/or VEGF to (A+B) 10.000 and (C+D) 17.500 cells/well of each cell type in the co-culture assay (HUVEC donor 1); amount of added growth factors: (A+C) 10 ng/ml PDGF-BB and (B+D) 40 ng/ml VEGF +10 ng/ml PDGF-BB; CD31 staining (endothelial cells, red)

**Figure 6:**
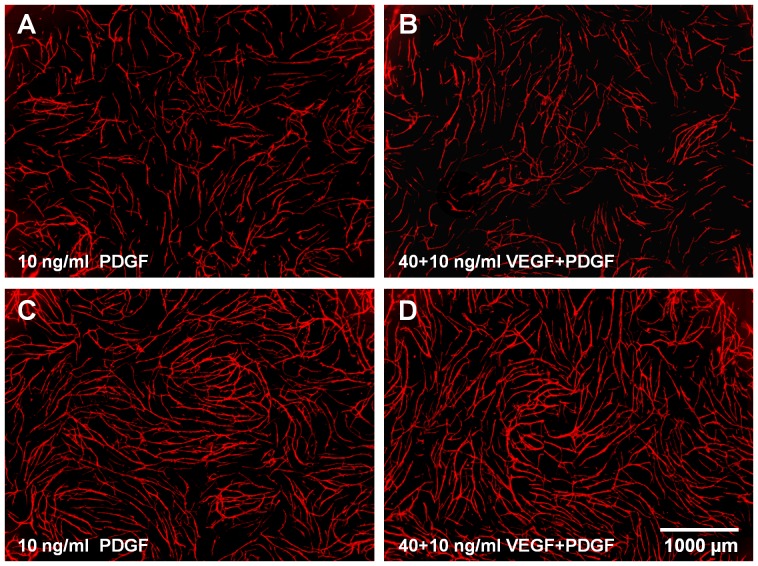
Effect of PDGF-BB and VEGF on HUVEC donor 2. Addition of PDGF-BB and/or VEGF to (A+B) 10.000 and (C+D) 17.500 cells/well of each cell type in the co-culture assay (HUVEC donor 2); amount of added growth factors: (A+C) 10 ng/ml PDGF-BB and (B+D) 40 ng/ml VEGF +10 ng/ml PDGF-BB; CD31 staining (endothelial cells, red)

**Figure 7:**
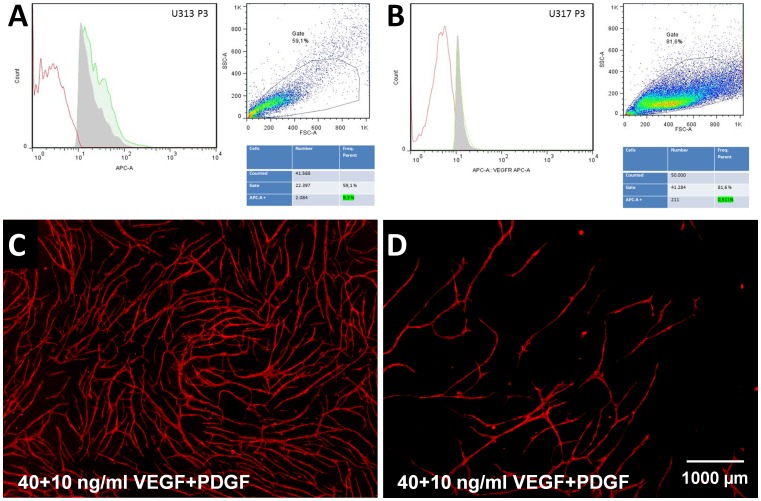
Correlation between FACS analysis of VEGFR-2 and formation of capillary-like structures. The correlation between (A+B) FACS analysis of VEGFR-2 and the (C+D) formation of capillary-like structures for two different donors in the co-culture assay; (A+C) Donor 1 (9.3% VEGFR-2) and (B+D) donor 3 (0.6% VEGFR-2); CD31 staining (endothelial cells, red)

**Figure 8:**
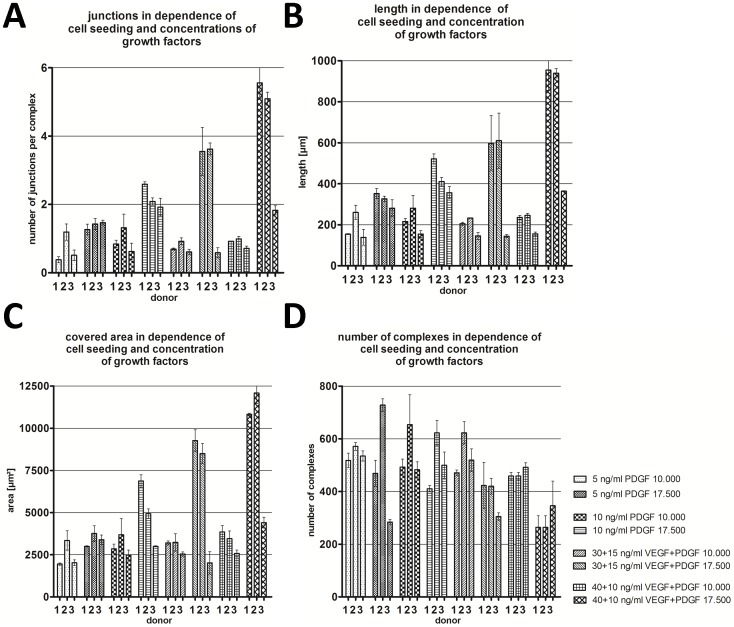
Quantification of capillary-like structures. (A) Junctions per complex, (B) length, (C) covered area and (D) number of complexes in the co-culture assay in dependence of cell seeding (10.000 or 17.500 cells/well for each cell type) and growth factor concentrations (5–15 ng/ml PDGF-BB and 30–40 ng/ml VEGF) for three different donors

The addition of pro-angiogenic factors, PDGF-BB (5–15 ng/ml) and/or VEGF-A (30–40 ng/ml), resulted in a higher amount of capillary-like structures for the 2 donors with the highest concentration of VEGFR-2 receptors (Figure 5,6,7). For these VEGFR-2-rich cell lines, the length, number of junctions and area were positively affected by the addition of both PDGF-BB and VEGF-A, with particular regard to the higher cell seeding number (1.75×10^4^) (Figure 8A–D). Considering the lower cell numbers, the effect of the additional growth factors was not significantly different. Regarding the 3^rd^ HUVEC donor, no positive effect was observed with the addition of low concentrations of VEGF and PDGF-BB, although the highest concentration used for PDGF-BB (10 ng/ml) and VEGF-A elicited a positive effect on the amount of capillary-like structures.

Regarding the different donor cells, HUVECs of different origin had an effect on the amount of capillary-like structures, with most capillary-like structures in the case of donors 1 and 2, compared with significantly less structures in the case of donor 3 (Figure 7).

**Figure 9:**
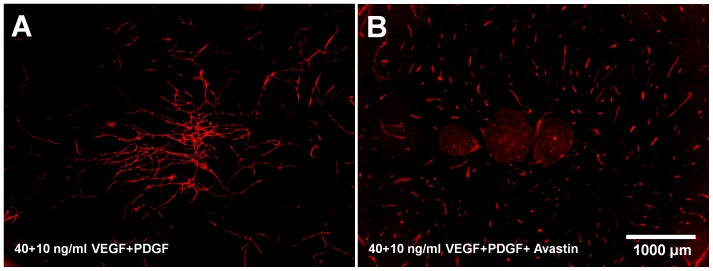
The effect of Bevacizumab on the formation of capillary-like structures. The effect of Bevacizumab on 17.500 cells/ well (each cell type) and 40+10 ng/ml VEGF+PDGF-BB; (A) without Bevacizumab and (B) with Bevacizumab; CD31 staining (endothelial cells, red)

The addition of Bevacizumab to the wells resulted in an inhibition of capillary-like structure formation (Figure 9). Fewer capillary-like structures were formed in the control wells employing donor 3 cells.

**Figure 10:**
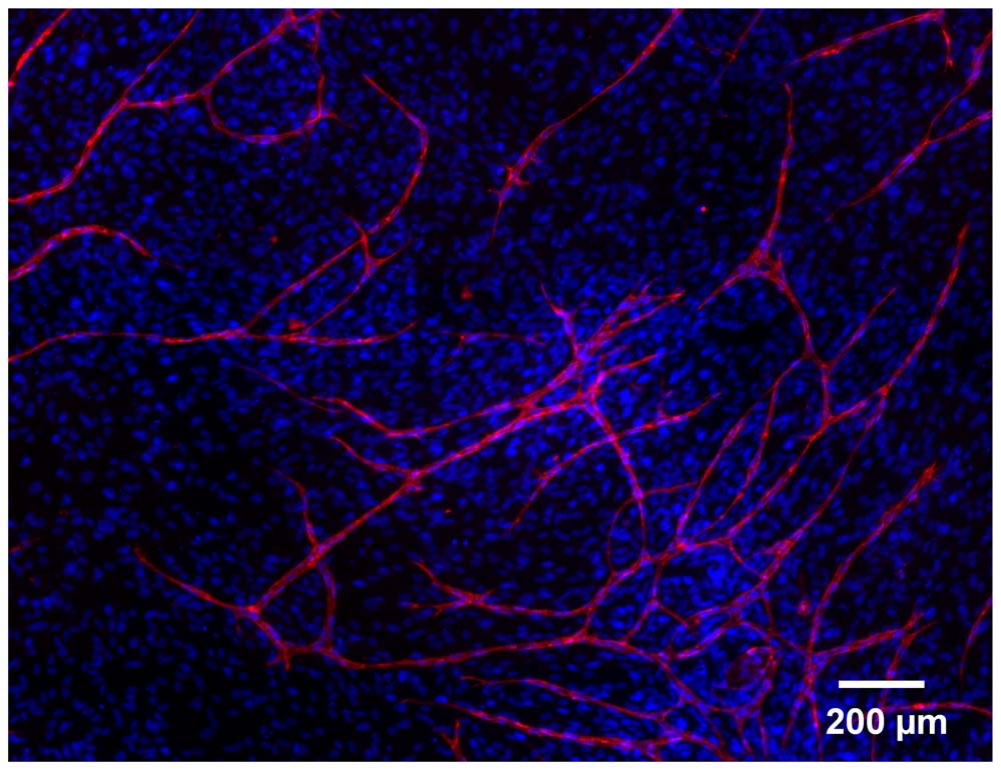
Co-culture assay with supportive ovine carotid-artery derived cells and HUVECs. DAPI (all cell nuclei, blue) and CD31 staining (endothelial cells, red)

The presence of the supportive cell line beneath and between the vessel-like structures could be demonstrated by DAPI staining (Figure 10).

### FACS analysis

The different HUVEC cell lines from various donors were examined for the presence of VEGFR-2 receptor. It was observed that the levels of VEGFR-2 expression were high in the cases of donor 1 (9.3%) and donor 2 (36.5%), with low levels of expression (0.5%) in the case of donor 3 (Figure 7).

### Immunoprecipitation

**Figure 11:**
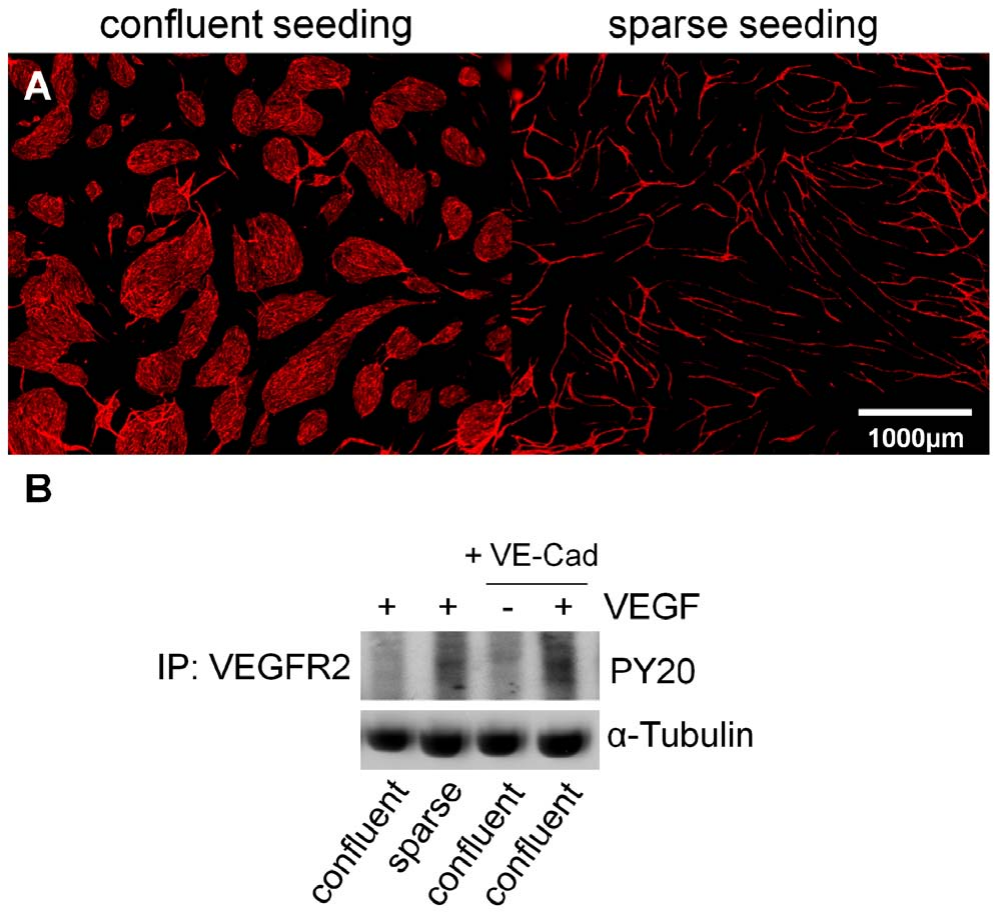
Correlation of VEGFR-2 with VE-Cadherin and capillary-like structures. (A) Confluent and sparse endothelial seeding in HUVEC/cartid artery-derived cell co-culture systems; CD31 endothelial cell staining; (B) Immunoprecipitated VEGFR2 blotted with anti-phosphotyrosine (PY 20) for detection of phosphorylated levels of VEGFR2; whole cell lysates were blotted with α-Tubulin to show equal loading.

The correlation between capillary-like structures, phosphorylated VEGFR-2 and VE-Cadherin has been demonstrated (Figure 11). The addition of VEGF to a sparse endothelial layer on ovine carotid artery-derived cells in a co-culture led to an increased amount of phosphorylated VEGFR-2 compared to the confluent endothelial layer. By adding VE-Cadherin antibodies and VEGF to the co-cultures with a confluent endothelial layer, the phosphorylation of VEGFR-2 increased compared to the VE-Cadherin only.

## Discussion

Blood vessels function to supply all cells of an organism with oxygen and nutrition. A tumour located in close proximity to this system and not exceeding a certain size (1–2 mm) is sufficiently supplied by diffusion. A larger tumour needs to recruit additional blood vessels by angiogenesis. The tumour itself can initiate these processes by releasing growth factors such as VEGF [1], and by consequently changing the balance between pro- and anti-angiogenic molecules. This results in pathological angiogenesis in the direction of the tumour. In order to limit tumour growth and metastasis, angiostatic drugs can be administered to inhibit the development of blood vessels and the nutrition of the tumour. In this case, the different stages of angiogenesis can be a target for drug administration: cell migration, proliferation and differentiation. Since the differentiation stage of angiogenesis is the final and most important step during which capillary-like tubules are formed, the assays simulating this stage are extensively used to determine the efficacy of anti-angiogenic drugs.

Currently used differentiation assays involve the plating of endothelial cells into or onto a gel matrix (collagen, fibrin or Matrigel) to investigate the formation of tubule-like structures after 4 to 24 h. The Matrigel assay is a widely used differentiation assay, where a protein mixture derived from mouse Engelbreth-Holm-Swarm sarcoma is used. After 12 h of endothelial cell seeding on the Matrigel, the cells start to form cord-like structures, although in many cases, the seeded cells often clump into cell aggregates [6]. Additionally, there is significant debate as to whether these cord-like structures actually contain patent lumina [4] or not [5]. Furthermore, non-endothelial cells like fibroblasts and glioblastoma cells have also been shown to form cord-like structures on Matrigel [6], and therefore a cautious interpretation of the results in this system is required. A further limitation of these gel assays is their inability to mimic the complete *in vivo* situation, since the surrounding cells near an angiogenic site are not taken into consideration.

In order to test angiostatic drugs efficiently, definitive *in vitro* assays are necessary to reliably estimate the anti-angiogenic potential of the drugs. 2-D assays should be fast, inexpensive and easy to set up. Furthermore, the assays should optimally imitate the *in vivo* situation. Most translatable assays would include supporting cells (e.g. smooth muscle cells, pericytes, and/or fibroblasts), extracellular matrix (ECM) and/or basement membrane. Circulating blood would further optimize an *in vitro* assay [12]. After an estimation of applicability via the 2-D assay, an *in vivo* assay could then be considered for further analysis.

Co-culture models could imitate the *in vivo* situation in a more reliable manner than Matrigel or other ECM models, since supportive cells adjacent to the angiogenic site in the body also have an influence on vessel development. Several co-culture models have been described so far, using HUVECs or human dermal microvascular endothelial cells (HDMVECs) as sprout-building cells, and different supportive cell types in 2D and 3D. These supportive cells develop an ECM [13] and/or release growth factors that support angiogenesis [14]. As supportive cells in co-culture models, fibroblasts from dermal human breast tissue, HUASMCs [7], pulmonary artery smooth muscle cells, mesenchymal stem cells [8] and fibroblasts from dermal superficial skin layer [9] have been used. All of these supportive cells in co-culture with sprout-forming cells supported the development of capillary-like structures in 2D. These co-cultures provide a reliable estimation regarding the anti-angiogenic potential of a particular drug. Unfortunately, all supportive cells used in the aforementioned assays derive from human tissue cell sources with limited access.

In this study, we developed a co-culture system employing ovine carotid artery-derived cells as supportive cells for angiogenesis. Although these cells are of animal origin, the formation of capillary-like structures was repeatable for different HUVEC sources, as well as for different supportive ovine carotid-derived cell lines. Furthermore, the availability of these supportive cells is superior in comparison to human cell sources. Typically in most laboratories, cells of human origin are more difficult to obtain due to donor shortage.

The formation of capillary-like structures by HUVECs on supportive ovine carotid-artery derived cells was demonstrated for different donors after 9 days (n = 6). After 4 days, HUVECs began to form capillary-like structures on the supportive ovine cells. It was observed that the exchange of the supportive cell line always enabled the formation of capillary-like structure formation (not shown). The presence of the supportive cell line beneath and between the vessel-like structures could be demonstrated by DAPI staining (Figure 10).

Regarding the cell number, 1.75×10^4^ supportive and endothelial cells resulted in the most extensive vessel networks with regard to length, number of junctions and area. Co-cultures with fewer cell numbers (1×10^4^ per cell type) formed less extensive structures, while co-cultures with larger cell numbers (2×10^4^ per cell type) promoted the formation of confluent mats of endothelial cells, rather than vessel structures. Cell numbers described in the literature were distinct from those employed in this assay, partly due to the use of 96-well plates [8], [9]. One explanation for the formation of less capillary-like structures with a higher number of supportive and endothelial cells (2×10^4^ per cell type) could be that the endothelial cells form a cobblestone monolayer from the outset, and adhere to the borders of the well directly. In published literature concerning monolayer cultures, it has been demonstrated that confluent HUVECs respond poorly to proliferate signals of VEGF and other growth factors [15], [16]. Additionally, this phenomenon seems to be directly linked to VEGFR-2 phosphorylation via VE-cadherin [15]. It seems that contact inhibition of endothelial cells has an influence on the amount of VE-cadherin controlling the VEGF-induced VEGFR-2 phosphorylation and therefore its activation. In our studies, we have proven that there is a correlation between the cell number, the amount of phosphorylated VEGFR-2 and the amount of capillary-like structures. Adding VE-Cadherin antibody to the culture 1 h before adding VEGF (5 min, 40 ng/ml) as a stimulus resulted in more phosphorylation of VEGFR-2 compared to the control. This indicates that there is a correlation between VE-Cadherin, phosphorylated VEGFR-2 and capillary-like structures. It seems that VE-Cadherin in a confluently seeded endothelial cell layer regarding a co-culture system with carotid artery-derived cells inhibits the phosphorylation of VEGFR-2 and therefore its activation, resulting in less capillary-like structure formation, as already shown for endothelial monocultures [15].”

The addition of pro-angiogenic factors, PDGF-BB (5–15 ng/ml) and/or VEGF-A (30–40 ng/ml), resulted in a higher amount of capillary-like structures for HUVEC donors with higher concentrations of VEGFR-2 receptors. For the third donor, exhibiting a low concentration of VEGFR-2 receptors, the amount of capillary-like structures could not be increased. Only the addition of 10 ng/ml PDGF-BB and 40 ng/ml VEGF resulted in an increase of capillary-like structures. It seems that VEGF-A combined with low concentrations of PDGF-BB has no effect on the amount of capillary-like structure formation on HUVEC cell lines with a low concentration of VEGFR-2 receptors, whereas 10 ng/ml PDGF-BB combined with VEGF-A causes an increase in capillary length, number area and complexes. A further significant increase in capillary-like structures could not be promoted using 30 ng/ml VEGF and 15 ng/ml PDGF-BB. In the literature, concentrations greater than 10 ng/ml PDGF-BB have not been reported for *in vitro* co-culture models [17], [18].

For the HUVEC cell lines with a high concentration of VEGFR-2 receptors, the length, number of junctions, and area were positively influenced by the additional growth factors. This positive effect of VEGF supplementation on capillary-like structure formation correlates with the findings observed in other co-cultures [6], [7] as well as in *in vitro* and *in vivo* models of ischemia [19], [20]. The cell morphology did not change in either supportive or vessel-forming cell monolayer cultures by addition of VEGF or PDGF-BB in the aforementioned concentrations (not shown).

In the case of PDGF-BB, a concentration of 5 ng/ml was shown to increase the length, number of junctions and area for donors with a high concentration of VEGFR-2 receptors, whereas 10 ng/ml had no additional significantly positive effect. PDGF-BB is an important growth factor involved in vascular development, as well as the development of kidney, lungs and the central nervous system [21]. Although PDGF-BB is not involved in the initial stages of angiogenesis, it stabilises new vessels, induces anastomosis and recruits pericytes to the neovessel. It has been shown that it is strongly expressed *in vivo* by endothelial tip cells for the recruitment of supportive cells [22]. PDGF has also been used in other 2D co-culture systems where indirect or direct positive effects on capillary network formation could be observed [17], [18]. However, the cell types, cell numbers and media employed in these studies were different to those used in the present study. In the co-culture systems, it can be speculated that the positive effect of PDGF-BB is accomplished by increasing VEGF expression levels in mural cells [23] and fibroblasts [24]. Additionally, it stimulates the production of collagenases, which are important for cell migration [25].VEGF and PDGF-BB have been used in sustained release systems both *in vitro* and *in vivo*, leading to a rapid and sustained increase of a mature vascular system [26], [27], [28].

The combination of the two different growth factors VEGF-A and PDGF-BB in two different ratios was applied to evaluate their effect on the amount of capillary-like structures. Although it is well known that both of these factors have a pro-angiogenic effect, it has not been investigated whether or not there may be a synergistic angiogenic effect of both factors in the co-culture system. In the present investigation, it could be demonstrated that there is a measurable synergistic effect of both growth factors for HUVEC cell lines with a high concentration of VEGFR-2 receptors.

Considering the lower cell numbers, the effect of VEGF and/or PDGF-BB supplementation was not significantly different. It seems that the effect of VEGF-A/PDGF-BB on the formation of capillary-like structures in the proposed co-culture system can only be recognized in the system where a higher concentration of cells is used. In the experiments with lower cell numbers, all endothelial cells already stretched to resemble capillary-like structures, with no further endothelial cells remaining to be stimulated to form additional capillary-like structures. In case of a higher cell seeding number, there are more capillary-forming endothelial cells in a cobblestone pattern which can be stimulated via VEGF-A/PDGF-BB to reorganize into capillary-like structures. In this case, the difference is measureable.

Regarding the 3^rd^ donor, the positive effect by adding VEGF to PDGF-BB was only statistically different at concentrations of 40 ng/ml VEGF and 10 ng/ml PDGF-BB.

In the course of this study, it was observed that the presence of VEGFR-2 receptors on HUVECs correlates with the number of capillary-like structures obtained in the endothelial co-culture assay. A lower expression of VEGFR-2 on HUVEC cells (donor 3 with 0.5%) compared to higher levels of expression in donor 1 (9.3%) and donor 2 (36.5%) correlated to a decrease in length, number of junctions and area of the capillary-like structures, and it is clearly evident that the cells with low concentrations of VEGFR-2 receptors are less responsive to stimulation by VEGF-A. With regard to VEGFR-2 expression and its activity in combination with different supportive cell numbers, the increases in length and number of junctions correlated with increasing numbers of supportive cells for all donors. With regards to the 3^rd^ donor, however, the increase in both length and number of junctions in the presence of increasing numbers of supportive cells was lower compared to the other two donors. This limitation may be explained by the lower concentrations of VEGFR-2, and by the fact that more supportive cells lead to improved ECM development resulting in enhanced endothelial cell attachment to the supportive layer.

VEGFR-2 is a critical VEGF receptor during development and adult angiogenesis. It is well accepted that VEGFR-2 is the most important mediator for mitogenic, angiogenic and permeability-enhancing effects of VEGF on endothelial cells [29]. In the literature, it has been shown that VEGFR-2 knockdown completely inhibits network formation in a 2D co-culture system [8]. The inhibition of VEGFR-2 led to similar results in a 3D co-culture model [30].

With respect to the testing of an anti-angiogenic drug in the presented co-culture system, the negative effect of Bevacizumab on the formation of capillary-like structures could be demonstrated. The effect was investigated for donor 3 with the addition of both 40 ng/ml VEGF and 10 ng/ml PDGF-BB. In this case, the amount of capillary-like structures decreased. Bevacizumab is an FDA-approved VEGF antibody which binds VEGF-A and proteolytic fragments, thereby inhibiting the development of blood vessels [3]. In co-culture as well as in *in vivo* models, this effect has also been demonstrated by other groups [31], [32]. In other co-culture experiments, suramin or curcumin have been successfully used as anti-angiogenic drugs [7].

The presently introduced co-culture system of ovine carotid-derived supportive cells and HUVECs demonstrates that the effect of pro- and anti-angiogenic effects on capillary network development can be measured and quantified in an easy and reliable manner. However, donor variability as a possible limitation in the endothelial co-culture assay needs to be taken into account. The supportive cells from ovine origin offer a less limited cell source compared to human cell lines. Furthermore, this system works in 96-well plates, consuming both fewer cell numbers and drug quantities in contrast to other systems. With this system, the effects of pro- and anti-angiogenic factors on individual HUVEC cell lines can be conveniently analysed. Since the patient's own cells could be potentially be used for this assay, the effect of an anti-angiogenic treatment, for example, can be routinely evaluated *in vitro*.
